# Synthesis of a Flexible Freestanding Sulfur/Polyacrylonitrile/Graphene Oxide as the Cathode for Lithium/Sulfur Batteries

**DOI:** 10.3390/polym10040399

**Published:** 2018-04-03

**Authors:** Huifen Peng, Xiaoran Wang, Yan Zhao, Taizhe Tan, Zhumabay Bakenov, Yongguang Zhang

**Affiliations:** 1 School of Materials Science & Engineering, Research Institute for Energy Equipment Materials, Hebei University of Technology, Tianjin 300130, China; peng@hebut.edu.cn (H.P.); wxran18709@163.com (X.W.); 2 Synergy Innovation Institute of GDUT, Heyuan 517000, China; taizhetan@gdut.edu.cn; 3 Nazarbayev University, National Laboratory Astana, Institute of Batteries LLC, 53 Kabanbay Batyr Avenue, Astana 010000, Kazakhstan; zbakenov@nu.edu.kz

**Keywords:** lithium/sulfur battery, sulfur/polyacrylonitrile/graphene oxide, freestanding cathode

## Abstract

Rechargeable lithium/sulfur (Li/S) batteries have received quite significant attention over the years because of their high theoretical specific capacity (1672 mAh·g^−1^) and energy density (2600 mAh·g^−1^) which has led to more efforts for improvement in their electrochemical performance. Herein, the synthesis of a flexible freestanding sulfur/polyacrylonitrile/graphene oxide (S/PAN/GO) as the cathode for Li/S batteries by simple method via vacuum filtration is reported. The S/PAN/GO hybrid binder-free electrode is considered as one of the most promising cathodes for Li/S batteries. Graphene oxide (GO) slice structure provides effective ion conductivity channels and increases structural stability of the ternary system, resulting in excellent electrochemical properties of the freestanding S/PAN/GO cathode. Additionally, graphene oxide (GO) membrane was able to minimize the polysulfides’ dissolution and their shuttle, which was attributed to the electrostatic interactions between the negatively-charged species and the oxygen functional groups on GO. Furthermore, these oxygen-containing functional groups including carboxyl, epoxide and hydroxyl groups provide active sites for coordination with inorganic materials (such as sulfur). It exhibits the initial reversible specific capacity of 1379 mAh·g^−1^ at a constant current rate of 0.2 C and maintains 1205 mAh·g^−1^ over 100 cycles (~87% retention). In addition, the freestanding S/PAN/GO cathode displays excellent coulombic efficiency (~100%) and rate capability, delivering up to 685 mAh·g^−1^ capacity at 2 C.

## 1. Introduction

Most of the energy consumption in the world is dependent mainly on fossil fuels which may be lacking in the future. This has led to a large amount of environmental, economic and social issues. Hence, the renewable energy sources like solar, tidal, and wind energy are becoming key components in the future energy supply. Nevertheless, intermittency and randomness of these alternate energy sources restricts their application. Hence, low-cost electrical energy storage, such as secondary batteries operated at room temperature have been regarded as promising power storage systems. In recent years, due to the rapid technological development of electronic devices, electric vehicles, and other electronic equipment, there is an imminent need for electrical energy storage systems with high performance [1].

The rechargeable lithium/sulfur (Li/S) batteries are deemed to be the most prospective candidates among lithium secondary batteries because sulfur is environment friendly, low cost, and nontoxic, and has a high theoretical specific capacity and energy density [1,2]. However, the practical application of Li/S batteries are restricted because of the low utilization of the active material, low conductivity of sulfur, and the lithium polysulfide shuttle behavior [3,4,5,6].

Additionally, sulfur can be firmly fixed on the polyacrylonitrile (PAN) backbone by heating the mixture of S and PAN, resulting in a high specific capacity and a good cyclability of the composites [7,8]. To some extent, the sulfur/polyacrylonitrile (S/PAN) composite cathodes control the shuttle effect, because polyacrylonitrile (PAN) acts as the conducting matrix and the sulfur (S) forms a covalent bond with polyacrylonitrile. In order to further increase the sulfur content and for utilization of the active material, S/PAN can be incorporated with conductive media, such as graphene, porous carbon fibers, carbon nanotubes, carbon paper interlayers, and metal oxides, etc. [5,9,10,11,12], testifying the excellent electrochemical performance displayed by multi-composites as the electrode for Li/S batteries.

A large number of reported works showed that graphene can improve the capacity, rate, and cycling performance of lithium/sulfur (Li/S) batteries [8,13,14,15]. Graphene oxide (GO) has a one-atom-thick, two-dimensional nanosheet structure with high surface area which offers superior structural flexibility, excellent mechanical properties, and wonderful conductivity [14,16,17]. Additionally, the graphene oxide (GO) membrane was able to minimize the polysulfides’ dissolution and their shuttle, which was attributed to the electrostatic interactions between the negatively-charged species and the oxygen functional groups on GO [16]. Furthermore, these oxygen-containing functional groups, including carboxyl, epoxide, and hydroxyl groups, provide active sites for coordination with inorganic materials (such as sulfur) [18,19]. Insertion of S/PAN particles between the graphene oxide layers effectively prevents severe aggregation of the material. Furthermore, the graphene oxide (GO) membrane provides space to accommodate the electrode volume changes during cycling, and both of these are propitious for cycling stability [20].

Although utilization of sulfur has been improved in these composite materials, the addition of electrochemically inactive conductive agent (super-p carbon black, SP) and binders (polyvinyldifluoride, PVDF or polytetrafluoroethylene, PTFE) reduces the overall gravimetrical capacity of the sulfur composite cathode due to reduction in content of the electrochemically active component. Meanwhile, a traditional electrode material applied onto a current collector, results in further lowering of mass fraction of the active material [21]. Therefore, the self-freestanding electrode material without any binder or a current collector could observably improve the energy density of batteries [22,23,24]. Recently, sulfur-based electrodes without metallic current collectors and binders were reported, which were made of sulfur infiltrated activated carbon fiber cloth or paper [25,26], a few interlayers composed of multi-walled carbon nanotubes (MWCNTs) [24], or carbon nanofibers (CNF) [22,24]. Furthermore, CNT and graphene oxides (GO) are usually used as a supporting material for freestanding electrodes because of their excellent conductivity, good mechanical properties, large specific surface area and flexibility [27,28,29].

In the present work, we synthesized a freestanding sulfur/polyacrylonitrile/graphene oxide (S/PAN/GO) hybrid binder-free electrode by vacuum filtration from a mixture of S/PAN/GO suspensions. Graphene oxide is used as a supporting material in S/PAN/GO composite because of its good mechanical properties, excellent electrical conductivity, large specific surface area, and flexibility. The freestanding S/PAN/GO cathode successfully utilizes the synergistic effect of the conductive PAN matrix and the GO slices wherein, the ability of the former to covalently bind with sulfur, increases the sulfur utilization, and capability of the latter remarkably enhances conductivity and structural stability of the system.

## 2. Materials and Methods

### 2.1. Materials Preparation

Polyacrylonitrile (PAN) (Sigma-Aldrich, Shanghai, China) and sulfur (S) (100-mesh particle size powder, Sigma-Aldrich, Shanghai, China) were manually mixed for 15 min in a weight ratio of 1:4 and heat treated at 450 °C for 6 h in a tube furnace in an argon atmosphere to form the sulfur/polyacrylonitrile (S/PAN) composite. Then 1 g S/PAN particles were dispersed in 125 mL graphene oxide (GO) solution (deionized water as a solvent, 2 mg·mL^−1^, Sigma-Aldrich, Shanghai, China) through stirring for 2 h thereby forming the S/PAN/GO aqueous suspension with a weight ratio of S/PAN:GO = 4:1. Subsequently, S/PAN/GO aqueous suspension was filtered by a 0.22 mm pore sized polyvinylidene difluoride (PVDF) membrane (50 mm diameter, Shanghai Xingya, Shanghai, China) and dried at 50 °C for 6 h. The S/PAN/GO free-standing film was easily detached from the filter film, and cut into disks of 1 cm in diameter for utilization as cathode for Li/S batteries. The final sulfur loading density of S/PAN/GO electrode was about 2.5 mg·cm^−2^. Furthermore, sulfur powder was dispersed in GO solution (S:GO = 4:1) and stirred for 2 h to obtain S/GO aqueous suspension. Subsequently, the S/GO film was easily obtained from the same filtration procedure as in case of S/PAN/GO film preparation. The final sulfur loading density of the S/GO electrode was about 2.1 mg·cm^−2^. For comparison, a conventional S/PAN cathode on a carbon coated aluminum foil current collector (Guangzhou Lanxi, Guangzhou, China) was prepared and tested. The slurry of S/PAN cathode was prepared by mixing the synthesized S/PAN composite, super-P (SP) carbon black (Tianjin Xinglongtai, Tianjin, China) and polyvinylidene difluoride (PVDF, Arkema, Suzhou, China) at a weight ratio of 8:1:1 in 1-methyl-2-pyrrolidinone (NMP, Sigma-Aldrich, Shanghai, China) solvent. Afterwards, the as-prepared slurry was applied onto the carbon coated aluminum foil (Guangzhou Lanxi, Guangzhou, China) and vacuum dried at 50 °C for 10h. The final sulfur loading density was about 2.0 mg·cm^−2^.

### 2.2. Physical Characterization

The morphologies of the S/PAN and S/PAN/GO composite surface were investigated using scanning electron microscopy (S-4800 FESEM, Hitachi Limited, Tokyo, Japan). The structural analysis of S/PAN/GO, S/PAN, GO, S particles were done by powder X-ray diffraction (XRD, Cu-Ka, λ = 0.154056 nm, Bruker D8 Discover, Bruker, Karlsruhe, Germany) at 40 kV with the range of 10° to 80°. The transformations in the chemical structure of the S/PAN/GO and S/PAN composite were deduced by Fourier transform infrared spectroscopy (FTIR, Bruker Tensor 27, Bruker, Karlsruhe, Germany). The identification of functional groups present in S/PAN/GO composite was performed by using X-ray photoelectron spectroscopy (XPS, Al-Kα, Kratos AXIS Ultra DLD, Shimadzu, Tokyo, Japan).

### 2.3. Electrochemical Characterization

The electrochemical properties were measured through CR2025-type Li/S coin cells assembled in an argon-filled glove box (Ar% ≥ 99.9995) with S/PAN/GO, S/GO and S/PAN composite as the cathodes, in three separate measurements. The electrochemical performance was evaluated using lithium metal as the anode, a porous polypropylene membrane as the separator, and 1 M LiPF_6_ in a solvent mixture of ethylene carbonate/dimethylcarbonate (EC/DMC) (1:1 by volume) as the electrolyte. The galvanostatic charging/discharging properties were tested between 1 V and 3 V using a battery tester (Neware, Shenzhen, China).

## 3. Results and Discussion

**Figure 1:**
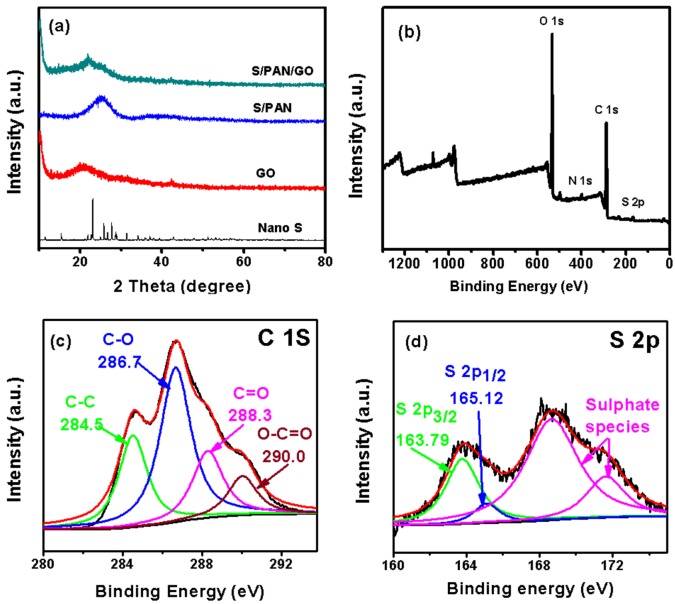
(**a**) XRD patterns of sulfur, sulfur/polyacrylonitrile (S/PAN), graphene oxide (GO), (sulfur/polyacrylonitrile/graphene oxide) S/PAN/GO; (**b**) XPS spectra and high-resolution spectra of (**c**) C 1s and (**d**) S 2p of the as-prepared sample.

The X-ray diffraction (XRD) patterns of sulfur (S), graphene oxide (GO), sulfur/polyacrylonitrile (S/PAN) and sulfur/polyacrylonitrile/graphene oxide (S/PAN/GO) composite are shown in black, red, blue, and green, respectively, in Figure 1a. In the pattern of S/PAN, the characteristic peaks of elemental sulfur disappear, indicating that fine sulfur particles exist in a highly dispersed state in the PAN matrix, and the crystal sulfur transforms into amorphous sulfur by the heat treatment. Compared to S/PAN, the XRD data of S/PAN/GO shows a new peak at 20° corresponding to the characteristic peak of graphene oxide, which proves that graphene oxide has been successfully added to the material (S/PAN/GO) [15,30,31]. The X-ray photoelectron spectroscopy (XPS) spectrum of S/PAN/GO is shown in Figure 1b, confirming the presence of O (535 eV), C (286 eV), and S (168 eV) elements. Furthermore, the C 1s spectrum of the S/PAN/GO (Figure 1c) has four peaks centered at 286.7, 284.5, 288.3, and 290.0 eV, which are attributed to C–O, C–C, C=O, and O–C=O bonds, respectively [32,33,34,35]. The S 2p peaks (Figure 1d) are attributed to the two forms of sulfur. The first peak comprises of two distinct peaks at 163.79 and 165.12 eV corresponding to the 2p3/2 and 2p1/2 of the –C–S– covalent bond [34,36]. The second peak (168.7–171.2 eV) corresponds to a sulphate or sulfonate species [37].

**Figure 2:**
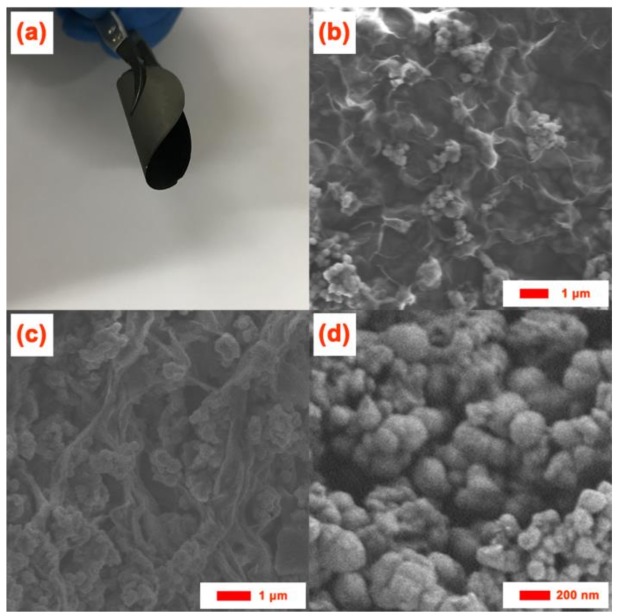
(**a**) Exhibition of S/PAN/GO composite with excellent flexibility; (**b**) SEM image of S/PAN/GO composite sample; (**c**) the longitudinal section image of S/PAN/GO films; and (**d**) SEM image of S/PAN composite sample.

Figure 2a demonstrates the flexibility and mechanical strength of the S/PAN/GO interlayer, which shows a full film of S/PAN/GO without any obvious defects in a bent state. The scanning electron microscopy (SEM) data were used for the analysis the microstructure of the samples. The morphologies of the S/PAN and freestanding S/PAN/GO composite cathodes are depicted in Figure 2b–d. In the image depicting the freestanding S/PAN/GO composite cathode (Figure 2b), S/PAN particles are evenly distributed among GO layers, keeping in close contact with it. The GO layer acts as both a conductive agent and binder for the S/PAN/GO composite cathode. The longitudinal section image of S/PAN/GO films (Figure 2c) shows that GO is entirely covered onto S/PAN composites, forming a conductive and mechanical protection for S/PAN composites, owing to graphene sheet structure. In Figure 2d, The S/PAN composite is revealed as a loose aggregation of S/PAN particles (100~200 nm).

**Figure 3:**
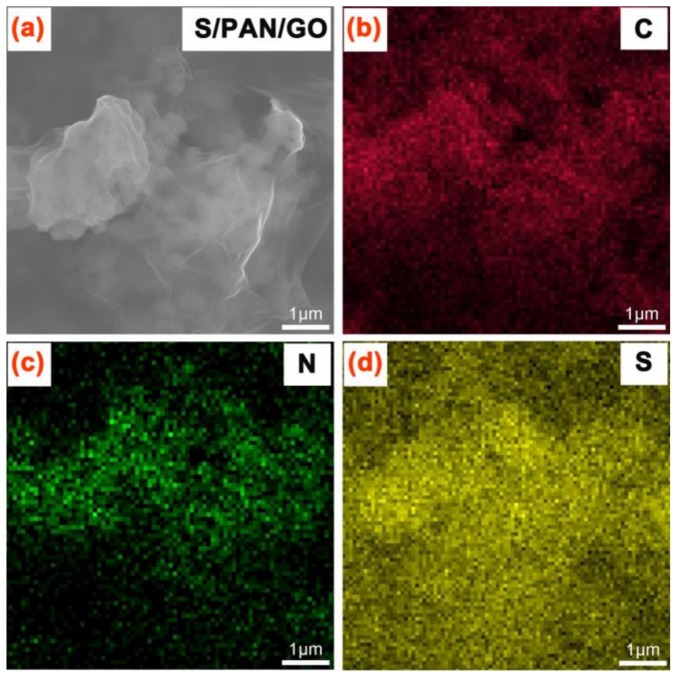
(**a**) SEM images of freestanding S/PAN/GO and element distribution of (**b**) C; (**c**) N; and (**d**) S.

Figure 3a displays the SEM images of freestanding S/PAN/GO. The S/PAN particles are covered with the GO layer. Figure 3b–d shows analysis of EDS elemental mapping, which clearly indicates the uniform distribution of components among the S/PAN/GO composite. In the Figure 3b–d, the mapping of carbon, nitrogen, and sulfur in the composite cathode indicate that sulfur is homogeneously distributed within PAN, and the S/PAN composite is uniformly mixed with GO.

**Figure 4:**
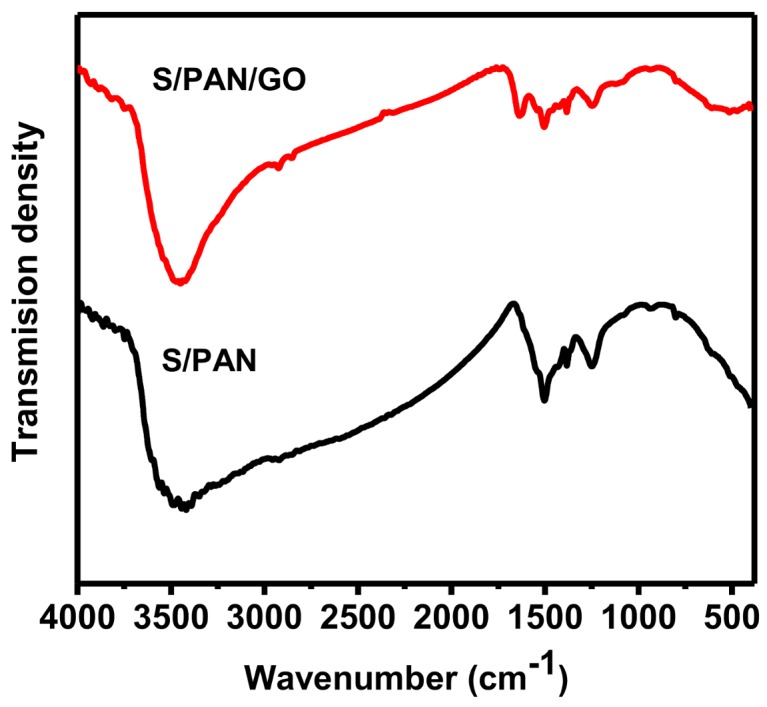
FTIR spectra of S/PAN and S/PAN/GO.

Figure 4 displays the FTIR spectra of S/PAN and S/PAN/GO samples. In the S/PAN and S/PAN/GO spectrum, many obvious peaks at the 1000 cm^−1^–1750 cm^−1^, corresponding to the covalent bond between sulfur and carbon in the polyacrylonitrile backbone, implies a successful reaction between sulfur and PAN [13]. Additionally, the characteristic peak at 1498 cm^−1^ corresponds to carbon–carbon double bond (C=C) and the medium peaks at 1428 cm^−1^ and 803 cm^−1^ correspond to the formation of the cyclic structure [8]. The characteristic peaks of the S/PAN composite are consistent with that of the S/PAN/GO composite, which indicates that the addition of GO has no effect on the chemical structure and properties of the S/PAN composite.

**Figure 5:**
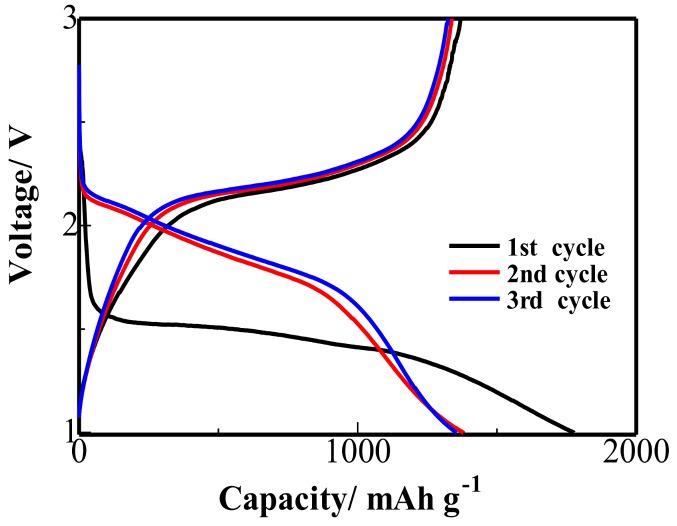
Discharge/charge profiles of Li/S batteries with freestanding S/PAN/GO cathode.

The profiles of galvanostatic discharge/charge tests of the S/PAN/GO cathode at a 0.2 C rate are depicted in Figure 5. The initial discharge capacity of the S/PAN/GO composite is 1776 mAh·g^−1^, which is much higher than the high theoretical specific capacity of sulfur (1675 mAh·g^−1^) owing to the irreversible insertion of lithium [13]. It can be seen that, the main plateau (1.3–1.6 V) appears in the discharge profile of the first cycle, which could be attributed to the key electrochemical reaction in Li/S battery. In the second and third cycle profiles, the reversible discharge capacity is 1379 and 1354 mAh·g^−1^ and the plateau appears at 1.7–2.2 V. Theoretically, a typical Li/S battery discharge curve should consist of two plateaus corresponding to a two-step reaction between lithium and sulfur. The first plateau, in the present case, was attributed to the reduction of the S_8_ ring and the formation of S_8_^2−^ and the second plateau was related to the reaction of Li_2_S*_n_* (4 ≤ *n* ≤ 8) to short-chain sulfide species (Li_2_S_2_ and Li_2_S) [38]. Nevertheless, voltage plateau of discharge profiles in the S/PAN/GO composites are different from those of elemental sulfur/carbon composite cathodes [25,26,29], which may result from the interaction between sulfur and PAN. The interaction between sulfur and PAN leads to the formation of a covalent bond between sulfur and carbon (S=C) in the PAN backbone, and this affects the interaction of sulfur with lithium. Additional energy is consumed to dissociate sulfur from the covalent bond (S=C), leading to a more negative potential [13,39].

**Figure 6:**
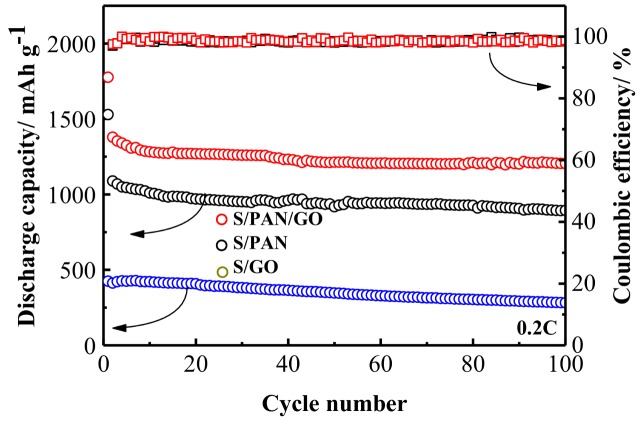
Cycling performance of the S/PAN, freestanding S/GO and freestanding S/PAN/GO cathodes.

Figure 6 displays the cycle performances of the S/PAN, S/GO, and S/PAN/GO composite cathodes at 0.2 C, respectively. The freestanding S/PAN/GO electrode delivers a high initial reversible discharge capacity of 1379 mAh·g^−1^, which is 290 mAh·g^−1^ higher than that of the S/PAN electrode and 950 mAh·g^−1^ higher than that of the S/GO electrode. It indicates that the sulfur/polyacrylonitrile (S/PAN) composite cathodes effectively curbed the shuttle effect and increased the utilization of active material. Graphene oxide (GO) successfully improved the conductivity of the electrode and enhanced the mechanical properties and structural flexibility for S/PAN/GO composite cathode. The S/PAN/GO composite cathode retains the discharge capacity of 1205 mAh·g^−1^ over 100 cycles, which accounts for ~87% capacity retention. In addition, the S/PAN/GO composite shows the average coulombic efficiency ~100% over 100 cycles, which could be due to suppression of the shuttle effect. However, S/PAN composite cathode maintains the reversible capacity of 894 mAh·g^−1^ after 100 cycles and the S/GO composite cathode retains the reversible capacity of 282 mAh·g^−1^.

**Figure 7:**
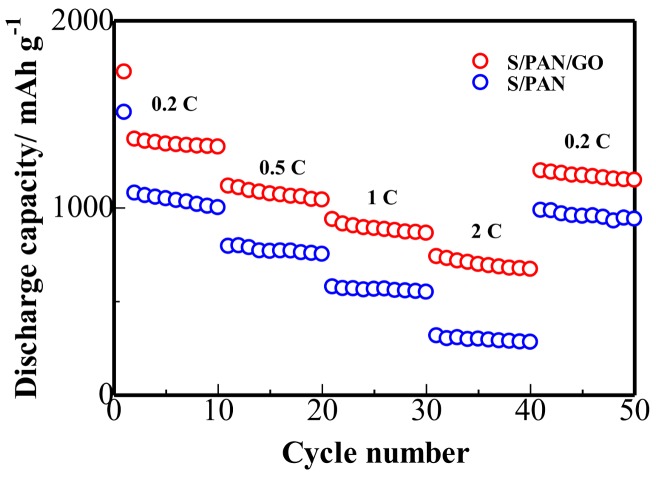
Rate capability of the S/PAN and S/PAN/GO electrodes at different c rates of 0.2 C, 0.5 C, 1 C, and 2 C.

The comparison on the rate performances of S/PAN and S/PAN/GO composite cathodes displayed in Figure 7, obviously demonstrates the advantages of the addition of GO, especially for a high-rate performance. The first discharge capacity of the S/PAN/GO composite cathode is 1776 mAh·g^−1^ at 0.2 C, which is 17% higher than that of the S/PAN electrode. The S/PAN/GO composite cathode delivers high capacities with different rates, namely, 1330 mAh·g^−1^ at 0.2 C, 1063 mAh·g^−1^ at 0.5 C, 872 mAh·g^−1^ at 1 C, and 685 mAh·g^−1^ at 2 C. Moreover, the discharge capacity can be recovered to 1161 mAh·g^−1^ when the rate is back to 0.2 C. In comparison to S/PAN/GO composite cathode; the discharge capacity of S/PAN electrode is lower for a different C rate. Furthermore, the composite S/PAN/GO electrode displays twice as much of the reversible capacity attained by the S/PAN electrode at a rate of 2 C. This indicates that the S/PAN/GO electrode has better conductivity, confirming the contribution of the highly-conductive graphene sheets towards the enhancement of the high rate performance.

## 4. Conclusions

In this paper, we reported the design and synthesis of a flexible freestanding sulfur/polyacrylonitrile/graphene (S/PAN/GO) ternary composite cathode via vacuum filtration. The application of novel S/PAN/GO film is an efficient way to improve electrochemical performance, which displays a high initial reversible discharge capacity of 1379 mAh·g^−1^ at 0.2 C and ~87% capacity retention tested over 100 cycles. In addition, the S/PAN/GO cathode delivers a higher coulombic efficiency (~100%). As a result, the excellent performance of freestanding S/PAN/GO composite cathode makes it a promising cathode for advanced Li/S batteries.

## References

[1] Zhou X., Xie J., Yang J., Zou Y., Tang J., Wang S., Ma L., Liao Q. (2013). Improving the performance of lithium–sulfur batteries by graphene coating. J. Power Sources.

[2] Sohn H., Gordin M.L., Regula M., Kim D.H., Jung Y.S., Song J.X., Wang D.H. (2016). Porous spherical polyacrylonitrile-carbon nanocomposite with high loading of sulfur for lithium–sulfur batteries. J. Power Sources.

[3] Lu S., Cheng Y., Wu X., Liu J. (2013). Significantly Improved Long-Cycle Stability in High-Rate Li–S Batteries Enabled by Coaxial Graphene Wrapping over Sulfur-Coated Carbon Nanofibers. Nano Lett..

[4] Nelson J., Misra S., Yang Y., Jackson A., Liu Y., Wang H., Dai H., Andrews J.C., Cui Y., Toney M.F. (2012). In Operando X-ray Diffraction and Transmission X-ray Microscopy of Lithium Sulfur Batteries. J. Am. Chem. Soc..

[5] Peng H., Wang X., Zhao Y., Tan T., Mentbayeva A., Bakenov Z., Zhang Y. (2017). Enhanced electrochemical performance of sulfur/polyacrylonitrile composite by carbon coating for lithium/sulfur batteries. J. Nanopart. Res..

[6] Yin F., Liu X., Zhang Y., Zhao Y., Menbayeva A., Bakenov Z., Wang X. (2017). Well-dispersed sulfur anchored on interconnected polypyrrole nanofiber network as high performance cathode for lithium–sulfur batteries. Solid State Sci..

[7] Zhang Y., Zhao Y., Bakenov Z., Konarov A., Chen P. (2014). Preparation of novel network nanostructured sulfur composite cathode with enhanced stable cycle performance. J. Power Sources.

[8] Doan T.N.L., Ghaznavi M., Zhao Y., Zhang Y., Konarov A., Sadhu M., Tangirala R., Chen P. (2013). Binding mechanism of sulfur and dehydrogenated polyacrylonitrile in sulfur/polymer composite cathode. J. Power Sources.

[9] Wang C., Chen J.J., Shi Y.N., Zheng M.S., Dong Q.F. (2010). Preparation and performance of a core–shell carbon/sulfur material for lithium/sulfur battery. Electrochim. Acta.

[10] Wei W., Wang J., Zhou L., Yang J., Schumann B., Nuli Y. (2011). CNT enhanced sulfur composite cathode material for high rate lithium battery. Electrochem. Commun..

[11] Yuan L., Yuan H., Qiu X., Chen L., Zhu W. (2009). Improvement of cycle property of sulfur-coated multi-walled carbon nanotubes composite cathode for lithium/sulfur batteries. J. Power Sources.

[12] Zheng W., Liu Y.W., Hu X.G., Zhang C.F. (2006). Novel nanosized adsorbing sulfur composite cathode materials for the advanced secondary lithium batteries. Electrochim. Acta.

[13] Li J., Li K., Li M., Gosselink D., Zhang Y., Chen P. (2014). A sulfur–polyacrylonitrile/graphene composite cathode for lithium batteries with excellent cyclability. J. Power Sources.

[14] Wang H., Yang Y., Liang Y., Robinson J.T., Li Y., Jackson A., Cui Y., Dai H. (2011). Graphene-wrapped sulfur particles as a rechargeable lithium–sulfur battery cathode material with high capacity and cycling stability. Nano Lett..

[15] Zhang Y., Zhao Y., Bakenov Z., Babaa M.R., Konarov A., Ding C., Chen P. (2013). Effect of Graphene on Sulfur/Polyacrylonitrile Nanocomposite Cathode in High Performance Lithium/Sulfur Batteries. J. Electrochem. Soc..

[16] Ji L., Rao M., Zheng H., Zhang L., Li Y., Duan W., Guo J., Cairns E.J., Zhang Y. (2017). Graphene Oxide as a Sulfur Immobilizer in High Performance Lithium/Sulfur Cells. J. Am. Chem. Soc..

[17] Wang Y.X., Huang L., Sun L.C., Xie S.Y., Xu G.L., Chen S.R., Xu Y.F., Li J.T., Chou S.L., Dou S.X. (2012). Facile synthesis of a interleaved expanded graphite-embedded sulphur nanocomposite as cathode of Li–S batteries with excellent lithium storage performance. J. Mater. Chem..

[18] Li D., Müller M.B., Gilje S., Kaner R.B., Wallace G.G. (2008). Processable aqueous dispersions of graphene nanosheets. Nat. Nanotechnol..

[19] Dong O.S., Park H., Lee Y.G., Kim K.M., Han T.H. (2014). Direct hybridization of tin oxide/graphene nanocomposites for highly efficient lithium-ion battery anodes. J. Electroceram..

[20] Chen H., Wang C., Dai Y., Qiu S., Yang J., Lu W., Chen L. (2015). Rational Design of Cathode Structure for High Rate Performance Lithium–Sulfur Batteries. Nano Lett..

[21] Zhao Y., Yin F., Zhang Y., Zhang C., Mentbayeva A., Umirov N., Xie H., Bakenov Z. (2015). A Free-standing Sulfur/Nitrogen Doped Carbon Nanotube Electrode for High Performance Lithium/Sulfur Batteries. Nanoscale Res. Lett..

[22] Qie L., Manthiram A. (2015). A Facile Layer-by-Layer Approach for High-Areal-Capacity Sulfur Cathodes. Adv. Mater..

[23] Zeng L., Pan F., Li W., Jiang Y., Zhong X., Yu Y. (2014). Free-standing porous carbon nanofibers-sulfur composite for flexible Li–S battery cathode. Nanoscale.

[24] Han S., Pu X., Li X., Liu M., Li M., Feng N., Dou S. (2017). High areal capacity of Li–S batteries enabled by freestanding CNF/rGO electrode with high loading of lithium polysulfide. Electrochim. Acta.

[25] Fu K., Li Y., Dirican M., Chen C., Lu Y., Zhu J., Li Y., Cao L., Bradford P.D., Zhang X. (2014). Sulfur gradient-distributed CNF composite: A self-inhibiting cathode for binder-free lithium–sulfur batteries. Chem. Commun..

[26] Swiderska-Mocek A., Rudnicka E. (2015). Lithium–sulphur battery with activated carbon cloth-sulphur cathode and ionic liquid as electrolyte. J. Power Sources.

[27] Zhu L., Peng H., Liang J., Huang J., Chen C., Guo X., Zhu W., Li P., Zhang Q. (2015). Interconnected carbon nanotube/graphene nanosphere scaffolds as free-standing paper electrode for high-rate and ultra-stable lithium–sulfur batteries. Nano Energy.

[28] Chen Y., Lu S., Wu X., Liu J. (2015). Flexible carbon nanotube-graphene/sulfur composite film: Free standing cathode for high-performance lithium/sulfur batteries. J. Phys. Chem..

[29] Sun L., Li M., Jiang Y., Kong W., Jiang K., Wang J., Fan S. (2014). Sulfur Nanocrystals Confined in Carbon Nanotube Network As a Binder-Free Electrode for High-Performance Lithium Sulfur Batteries. Nano Lett..

[30] Konarov A., Gosselink D., Doan T.N.L., Zhang Y., Zhao Y., Chen P. (2014). Simple, scalable, and economical preparation of sulfur–PAN composite cathodes for Li/S batteries. J. Power Sources.

[31] Liu Y., Zhao X., Chauhan G.S., Ahn J.H. (2016). Nanostructured nitrogen-doped mesoporous carbon derived from polyacrylonitrile for advanced lithium sulfur batteries. Appl. Surf. Sci..

[32] Meng Z., Xie Y., Cai T., Sun Z., Jiang K., Han W.Q. (2016). Graphene-like g-C3N4, nanosheets/sulfur as cathode for lithium–sulfur battery. Electrochim. Acta.

[33] Sun D., Ban R., Zhang P.H., Wu G.H., Zhang J.R., Zhu J.J. (2013). Hair fiber as a precursor for synthesizing of sulfur and nitrogen-co-doped carbon dots with tunable luminescence properties. Carbon.

[34] Li Y., Cai Q., Wang L., Li Q., Peng X., Gao B., Huo K., Chu P.K. (2016). Mesoporous TiO_2_ Nanocrystals/Graphene as an Efficient Sulfur Host Material for High-Performance Lithium–Sulfur Batteries. ACS Appl. Mater. Interfaces.

[35] Wang Z., Dong Y., Li H., Zhao Z., Wu H., Hao C., Liu S., Qiu J., Lou X.W. (2014). Enhancing lithium–sulphur battery performance by strongly binding the discharge products on amino-functionalized reduced graphene oxide. Nat. Commun..

[36] Lim V.W.L., Li S., Kang E.T., Neoh K.G., Tan K.L. (1999). In situ XPS study of thermally deposited aluminum on chemically synthesized polypyrrole films. Synth. Met..

[37] Wang X., Zhang Z., Qu Y., Lai Y., Li J. (2014). Nitrogen-doped graphene/sulfur composite as cathode material forhigh capacity lithium–sulfur batteries. J. Power Sources.

[38] Mikhaylik Y.V., Akridge J.R. (2004). Polysulfide Shuttle Study in the Li/S Battery System. J. Electrochem. Soc..

[39] Wang J., Yang J., Wan C., Du K., Xie J., Xu N. (2003). Sulfur Composite Cathode Materials for Rechargeable Lithium Batteries. Adv. Funct. Mater..

